# Ultrasound diagnostic of mesonephric paraovarian cyst - case report

**Published:** 2016

**Authors:** RE Bohîlțea, MM Cîrstoiu, N Turcan, CA Ionescu

**Affiliations:** * “Carol Davila” University of Medicine and Pharmacy, Bucharest, Romania; ** University Emergency Hospital Bucharest, Romania; *** ”Sf. Pantelimon” Clinical Emergency Hospital, Bucharest, Bucharest

**Keywords:** adnexal pathology, paraovarian cyst, ultrasound diagnostic, laparoscopy

## Abstract

Paraovarian cysts are a rare pathology, constituting 10-20% of the adnexal masses. The origin can be represented by paramesonephric ducts (Hydatid cysts of Morgagni), vestiges of mesonephric ducts also represented by mesothelium, or neoplastic (cystadenomas or cystadenofibromas) that are mostly benign. Borderline or malignant paraovarian tumors are encountered less often. This article presents a case of paraovarian cyst in a 37-year-old patient, with a history of 2 pregnancies, completed by cesarean. The patient sought medical attention for an asymptomatic voluminous ovarian cyst, detected in a routine ultrasound scan. Laboratory tests and tumor markers were within normal limits. Transvaginal ultrasound and color Doppler revealed a cystic adnexal mass with 10 cm transonic, smooth, homogeneous content, avascular walls with no internal papillary projections, with a “hyperechoic line” sign of delimitation from the ovarian capsule, mostly visible when the adnexa was mobilized. The diagnostic and curative laparoscopic surgery was successful, followed by a quick recovery. The histopathological exam confirmed the benignity and the origin of the paraovarian cyst. The case was discussed in the context of the literature review concerning this pathology, drawing attention to the real possibility of differentiating ovarian from paraovarian cysts by ultrasound.

## Introduction

Paraovarian cysts are found in the broad ligament between the ovary and the fallopian tube [**1**]. The cases have been reported in all female age groups and seemed to be most common in the third to fifth decades of life. The origin of these pelvic masses may be non-neoplastic, simple cyst or neoplastic [**2**]. Paraovarian simple cyst originates from the embryologic remnants of the urogenital system (mesonephric or Wolffian and paramesonephric or Mullerian ducts) [**1**]. In embryonic life, mesonephric and paramesonephric ducts are characteristic for both sexes. In female embryos, paramesonephric ducts rise to the main genital organs. It is possible that some portions from the cranial and caudal segments of the excretory tubules to persist in the female fetus in the mesovarium, forming the epoophoron and paroophoron. The mesonephric duct disappears, except for one small cranial segment located at the epoophoron level, and sometimes except for one small caudal segment that may remain in the uterus or the vaginal wall. A paraovarian cyst may be formed from these structures during the lifetime [**3**]. Another origin can be represented by the mesothelium, resulted from the invagination of the tube’s serosa. A simple cyst can suffer multiple transformations, resulting in a neoplastic paraovarian cyst, that is usually benign, or in a serous cyst similar to benign ovarian tumor (cystadenomas or cystadenofibromas). Borderline or malignant paraovarian tumors are encountered less often [**5**]. 

The reported incidence of malignancy is of about 2–3% [**6**]. In a review of 79 female patients with paratubal cysts, the paramesonephric variant was the most commonly encountered [**7**]. Paraovarian cysts constitute about 10-20% of the adnexal masses, and previously had a lower reported rate. A study from Italy [**2**] estimated their incidence to be of about 3%, while an autopsy study of postmenopausal women detected them in about 4% of the cases [**4**]. They are not uncommon, but due to their frequent asymptomatic presence, the actual incidence is not known. 

The symptoms can occur in the case of a gigantic size, or in the case of complications, such as hemorrhage, rupture or torsion or inflicting acute pain. Most cysts are small and asymptomatic, the reported sizes are 1 to 8 cm diameter, but larger lesions may reach 20 cm or more. Paraovarian cysts are often diagnosed intraoperatory, during routine imaging investigations or another disease management.

A simple, asymptomatic paratubal or paraovarian cyst can be managed expectantly without further follow-up. Surgical exploration and removal is indicated for these lesions, if they undergo torsion, cause persistent pain, pressure symptoms, or appear neoplastic with suspicious findings by ultrasonography (septations, papillations, fluid, and solid components). Laparoscopy is currently the most common surgical approach in the management of paraovarian cysts. Surgeons commonly use two techniques: the first includes the aspiration of the cystic fluid via the laparoscope, and the second includes the performance of a fenestration of the cyst before removing it. The risk of both described procedures is the spillage of a neoplastic cyst, with intraperitoneal dissemination of any existing malignant cells. Therefore, it is very important to differentiate the simple paraovarian cyst from the neoplastic paraovarian one, before deciding the surgical approach. The current data on the discrete characteristics of simple, versus neoplastic paraovarian cyst are limited. According to some studies [**8**], the risk of malignancy is higher when the size of the cyst is bigger than 5 cm, but other clinical or surgical differentiating criteria were not investigated. The preoperative ultrasound scan is mandatory intending to obtain defining information for a differential diagnosis between benign and malignant cysts, and on the other hand, between ovarian and paraovarian cysts. Unfortunately, no other different criteria than IOTA rules are available, despite the different origin of the paraovarian cysts. In order to establish the distinguishing criteria and for a proper diagnosis, computer tomography or magnetic resonance imaging may be performed in the preoperative evaluation, but none of these imaging techniques succeeded to provide a concrete result so far, and to precisely eliminate the misdiagnosis as an ovarian mass that remains to be a problem.

## Case report 

A 37-year-old woman presented an asymptomatic and voluminous ovarian cyst, detected during a routine ultrasound scan, one month before presentation. The personal history included menarche at 14 years old, regular periods and 2 pregnancies completed by cesarean. She denied having experienced weight loss, fever, chills, night sweats, urinary tract symptoms, or other gastrointestinal complaints.

**Fig. 1:**
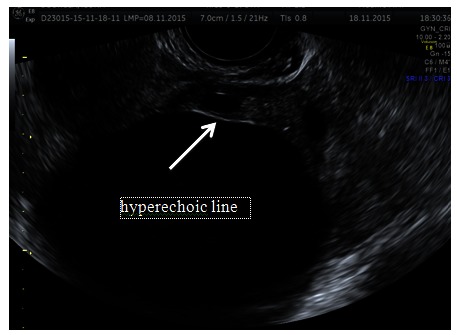
Conventional 2D transvaginal ultrasound evaluation of a paraovarian cyst; the hyperechoic line is always visible between the cyst and ovarian capsule when the cyst does not originate from the ovarian tissue

The clinical examination revealed a good physical appearance, weight of 55 kg and height of 160 cm. During a deep abdominal palpation, a voluminous adnexal mass with the upper pole corresponding to the umbilical scar was revealed. On pelvic exam, a smooth, round, rubbery mass, non-adherent to the surrounding tissues and no vaginal bleeding was found. Laboratory tests and tumor markers were within normal limits. Transvaginal ultrasound 2D and color Doppler revealed a cystic adnexal mass with 10 cm transonic, smooth, homogeneous content and avascular walls with no internal papillary projections, delimitated from the ovarian capsule by a “hyperechoic line” sign, being easier to establish the paraovarian origin by mobilizing the adnexa; a normal uterus and normal bilateral ovaries were described (**Fig. 1**). 

**Fig. 2:**
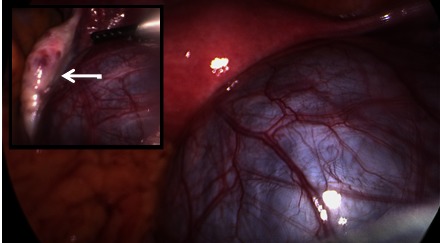
Laparoscopic view of the paraovarian cyst

The laparoscopic diagnostic and curative surgery (laparoscopic cystectomy) was indicated. The cyst had no attachments to the abdominal wall, intestine, or mesentery and it was successfully removed during the procedure (**Fig. 2**). The uterus, both ovaries and fallopian tubes were normal. Patient recovery was quick and uneventful.

**Fig. 3:**
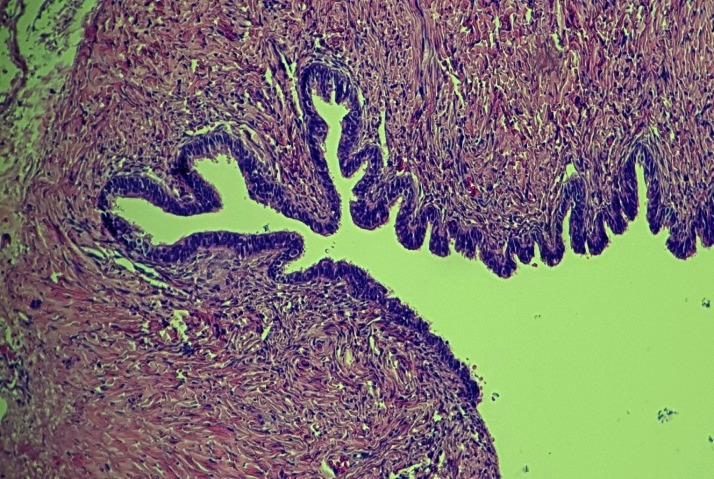
Laparoscopic view of the paraovarian cyst

**Fig. 4:**
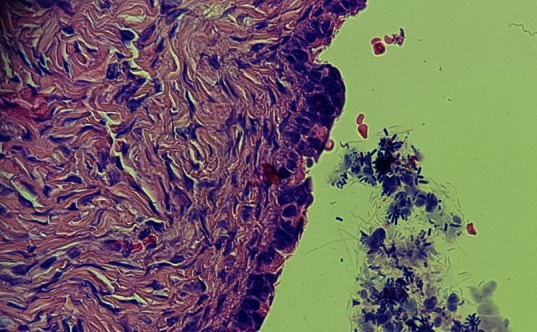
Fibrous connective tissue with

Microscopically, the paraovarian cyst wall was lined with the mesonephric epithelium, containing fibrous connective tissue with vascular structures and glandular epithelium, columnar and cuboidal at the periphery (**Fig. 3**, **4**). There was no evidence of malignancy, borderline epithelium, vascular malformation or other neoplasm. No ovarian stroma was found in any of the sections.

## Discussions and conclusions 

The optimal treatment of women with pelvic masses clinically suspected of being of adnexal origin requires knowledge of the exact nature of the mass. Ultrasound is considered the first-line diagnostic imaging tool for this task [**9**]. A thorough ultrasound examination can usually discriminate between benign and malignant pelvic tumors in the adnexal region. As an example, in the study of Sokalska et al. [**10**], the dermoid cysts, hydrosalpinges, functional cysts, paraovarian cysts, peritoneal pseudocysts, fibromas/ fibrothecomas and simple cysts were never misdiagnosed as malignancies by the ultrasound examiner [**11**]. The differential diagnosis between the ovarian cyst and the paraovarian ones remains difficult. In the literature, Barloon et al. [**12**] were able to correctly identify only one of ten paraovarian cysts by preoperative ultrasound examination, the remaining nine cysts being misdiagnosed as ovarian cysts or hydrosalpinx. They concluded that these masses are “difficult to diagnose before surgery at transabdominal and transvaginal sonography” and that “an ovarian cystic mass cannot reliably be differentiated from a paraovarian cyst”. Other authors [**13**] have reported similar results. In the study mentioned above [**9**], the endometriomas and dermoid cysts were confused, although very rarely, with a variety of other conditions (with no particular pathology being over-represented among the misdiagnoses). The serous cysts, adenofibromas, simple cysts, hydrosalpinx, functional cysts, and paraovarian/ parasalpingeal cysts were often confused with each other. This illustrates that many of the adnexal pathologies do not have a pathognomonic appearance at the ultrasound examination, including paraovarian cysts that are unfortunately often misinterpreted as true ovarian cysts, perhaps because the sonographic features have not been described in detail yet [**14**].

At the ultrasound exam, the paraovarian cyst appears as a round or oval well defined cystic mass, located close to the ipsilateral ovary, but clearly separated. The mobility and dissociation of the cyst from the ovary by the hyperechoic line is characteristic and defining for the diagnostic. The mobility of a paraovarian cyst depends on its size, position in the pelvis and anatomical relationship with the uterus and ovaries.

In the case presented in this article, the paraovarian cyst was not misdiagnosed during the ultrasound exam and was not confused with other pelvic tumoral masses. 
